# Untangling Geobacter sulfurreducens Nanowires

**DOI:** 10.1128/mbio.00850-22

**Published:** 2022-06-01

**Authors:** Derek R. Lovley

**Affiliations:** a Department of Microbiology and Institute for Applied Life Sciences, University of Massachusetts, Amherst, Massachusetts, USA; University of Illinois at Chicago

**Keywords:** *Geobacter*, e-pili, electromicrobiology, extracellular electron transfer, microbial nanowires

## LETTER

Ye et al. (1) present a model for how protein nanowires contribute to the current production of Geobacter sulfurreducens biofilms. They state that “In this study, we examined all three nanowires in the anode biofilm of G. sulfurreducens with the goal of defining both their structural and conductive contributions.” (1). However, Ye et al. (1) did not examine any nanowires in anode biofilms, and the only imaging they provided suggested that the strain of G. sulfurreducens that they studied did not express two of the three nanowire types under consideration. Furthermore, the authors misrepresent previously published studies of their own, as well as other investigators.

**FIG 1:**
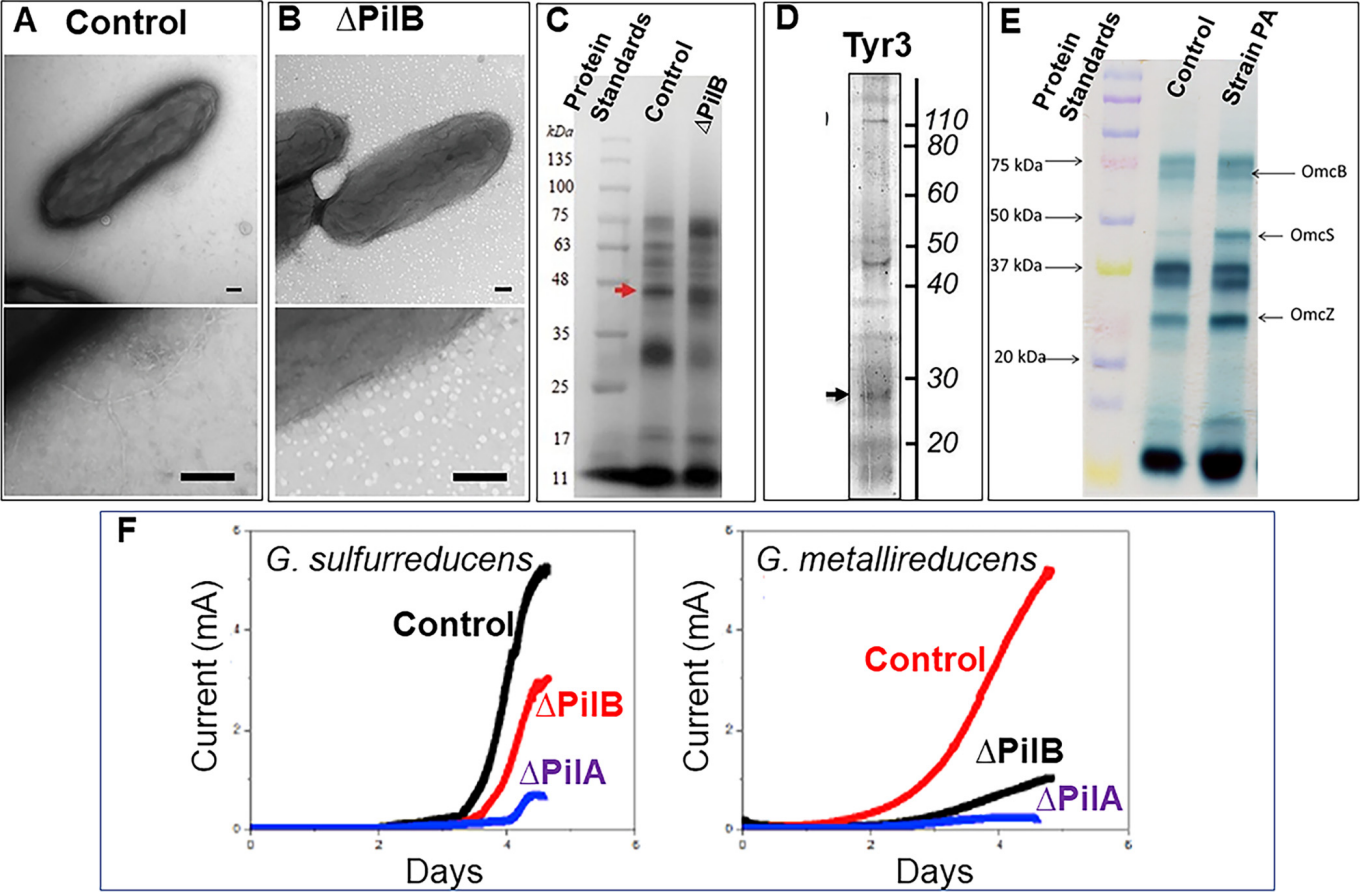
Data on the expression of nanowires and outer-surface cytochromes from previous studies. (A) Transmission electron micrographs of control strain of G. sulfurreducens from (1). Size bars are 100 nm. (B) Transmission electron micrographs of ΔPilB mutant of G. sulfurreducens from (1). Size bars are 100 nm. (C) Heme-stained SDS-PAGE of extracellular *c*-type cytochromes prepared from G. sulfurreducens control strain and ΔPilB mutant. The red arrow designates the band for OmcS. The gel image with an arrow is from reference (2) with permission. (D) Heme-stained proteins in the biofilm matrix from strain Tyr3, a mutant in which the tyrosines in the pilin monomer were replaced with alanine. The arrow designates the band for OmcZ. The gel image with an arrow is reprinted from reference (8) (publisher permission was not needed). (E) Heme stained SDS-PAGE of outer surface *c*-type cytochromes from G. sulfurreducens control strain and strain PA, a strain expressing poorly conductive pili. The gel image with band labeling is from reference (7) (publisher permission was not needed). (F) Current production of G. sulfurreducens and G. metallireducens control strains, ΔPilB strains, and ΔPilA strains. The data image is from reference (2) with permission. Descriptive labeling on original images was modified for consistency.

The three nanowires that Ye et al. (1) considered were electrically conductive pili (e-pili) and nanowires comprised of chains of multi-heme *c*-type cytochromes, either OmcS or OmcZ (1). Ye et al. (1) demonstrated that, as expected, their control strain of G. sulfurreducens expressed thin filaments (Fig. 1A). However, the resolution of their images was insufficient to determine whether the filaments were e-pili or cytochrome-based filaments. When Ye et al. (1) deleted the gene for PilB, no filaments were apparent (Fig. 1B). According to Ye et al. (1), deleting the PilB gene specifically eliminated the expression of e-pili. If this was true, then the filaments emanating from the control strain must have all been e-pili because they are all absent in the ΔPilB strain. The lack of filaments emanating from the ΔPilB strain could not be attributed to a lack of cytochrome expression because the same lab (2) previously demonstrated proper expression of outer-surface *c*-type cytochromes in their ΔPilB strain under the same growth conditions (Fig. 1C). Thus, not only did Ye et al. (1) fail to document that their strain of G. sulfurreducens produced cytochrome-based filaments, but they also presented data that suggested that the outer-surface cytochromes that were expressed were not assembled into filaments.

Although OmcS nanowires emanating from cells grown under similar conditions were previously demonstrated in another strain of G. sulfurreducens (3), it is also known, as recently reviewed in detail in references (4, 5), that OmcS and OmcZ can be displayed on the outer cell surface without forming nanowires. Without first proving that their strain can express OmcS and OmcZ nanowires, Ye et al. (1) had no data to support their assertion that deleting genes for OmcS or OmcZ removed OmcS or OmcZ nanowires.

Previous studies concluded that e-pili plays an important role in long-range electron transport through current-producing G. sulfurreducens biofilms because strains expressing poorly conductive pili were defective in current production (6–10). Ye et al. (1) misrepresent these earlier studies by suggesting that expressing poorly conductive pili “is usually incurred at altering the extracellular cytochrome profile”. The reference they cite for this (8) stated the direct opposite conclusion and provided data that cytochromes were properly localized (Fig. 1D). Ye et al. (1) also ignored additional studies, including a previous publication by one of the authors (7), which demonstrated proper expression of outer surface cytochromes in strains expressing poorly conductive pili (Fig. 1E). The most recent study on this topic demonstrated that a strain of G. sulfurreducens that can express OmcS nanowires continued to produce OmcS nanowires in the same abundance when it was genetically modified to express poorly conductive pili (3). Ye et al. (1) needed to change their model to account for Liu’s prior results (7), as well as the results of other investigators, that have reported proper localization of outer surface cytochromes, but diminished current production in G. sulfurreducens strains expressing poorly conductive pili.

Liu et al. (2) previously reported that ΔPilB strains of G. sulfurreducens and G. metallireducens were defective in current production (Fig. 1F). Yet now, Ye et al. (1) claim that a ΔPilB strain produces ca. 90% as much current as wild-type. This reversal in the phenotype reported is remarkable because it has a major impact on the nanowire model. If, as the authors claim, deleting the gene for PilB specifically prevents e-pili expression, then the phenotype that their lab originally reported (2) refutes their claim that e-pili had a minor role in electron transfer through biofilms. An explanation for their change in the phenotype reported is required.

Ye et al. (1) presented a model in which OmcZ nanowires coursing throughout current-producing biofilms were the primary conduit for long-range electron transport. This conclusion was based on the finding that deleting the gene for OmcZ greatly diminished current production. Ye et al. (1) fail to note that this same phenotype was reported long ago (11) and that, based on those results, the localization of OmcZ was intensively investigated (12). Those studies demonstrated that OmcZ was specifically localized at the biofilm-anode interface and that OmcZ did not appear to be organized in filaments (12). Thus, the model of Ye et al. (1) was unfounded because they did not provide data to refute the earlier studies on OmcZ localization, and as noted above, Ye et al. (1) did not provide evidence that their strain of G. sulfurreducens could express the OmcZ nanowires emanating from cells that their model required.

Word limit restrictions prevent full discussion of the failure of Ye et al. (1) to properly present all the data, including contradictory data from their prior studies, that refute their model. They misrepresent recent studies (13) that questioned the rationale behind generating ΔPilB mutants by suggesting that those studies were conducted on a G. sulfurreducens strain not relevant to their strain without acknowledging that the prior studies also analyzed a ΔPilB mutation made in the type strain background. Ye et al. (1) also fail to properly attribute previous key discoveries which they imply are their own, such as the fact that e-pili can also play a structural role in biofilm formation (14), as well as the major importance of OmcZ and lesser role of OmcS in high-density current production and biofilm growth on anodes (11).
